# Heme handling in the system II heme lyase CcsBA from *Bacteroides thetaiotaomicron*

**DOI:** 10.1007/s00775-026-02171-y

**Published:** 2026-08-25

**Authors:** Julia Seifermann, Lorena Ilcu, Céline Moog, Lin Zhang, Oliver Einsle

**Affiliations:** 1 Institute of Biochemistry, Albert-Ludwigs-Universität Freiburg, Albertstrasse 21, 79104 Freiburg, Germany; 2 Faculty of Synthetic Biology, Shenzhen University of Advanced Technology, Shenzhen, 518107 China

**Keywords:** *C*-type cytochromes, Cytochrome *c* maturation, Heme lyase, cryo-electron microscopy, Metalloprotein assembly

## Abstract

Cytochrome *c* biogenesis in bacteria relies on complex membrane-bound machineries that facilitate the covalent attachment of heme to apocytochrome *c*. System II, mediated by the bifunctional protein CcsBA, is prevalent in Gram-positive and some Gram-negative bacteria, as well as chloroplasts. Here, we report a 2.9 Å resolution cryo-EM structure of a W703C mutant of CcsBA from *Bacteroides thetaiotaomicron*, revealing its conformation in the heme-loaded, closed state. Comparative analysis with the previously published *Helicobacter hepaticus* CcsBA structure shows a conserved membrane architecture and WxWD domain organization, but notable differences in the arrangement of the periplasmic domain and the active site configuration. The W703C mutation allowed heme occupancy in the active site without inducing the open conformation, implicating the native W703 in regulating structural transitions critical for heme attachment. Our findings suggest that while the open conformation facilitates heme ligation, it is not essential for heme translocation. This work expands the understanding of structure–function relationships in System II cytochrome *c* maturation and highlights the potential regulatory role of the periplasmic domain conformational dynamics.

**Supplementary Information:**

The online version contains supplementary material available at https://doi.org/10.1007/s00775-026-02171-y.

## Introduction

**Fig. 1:**
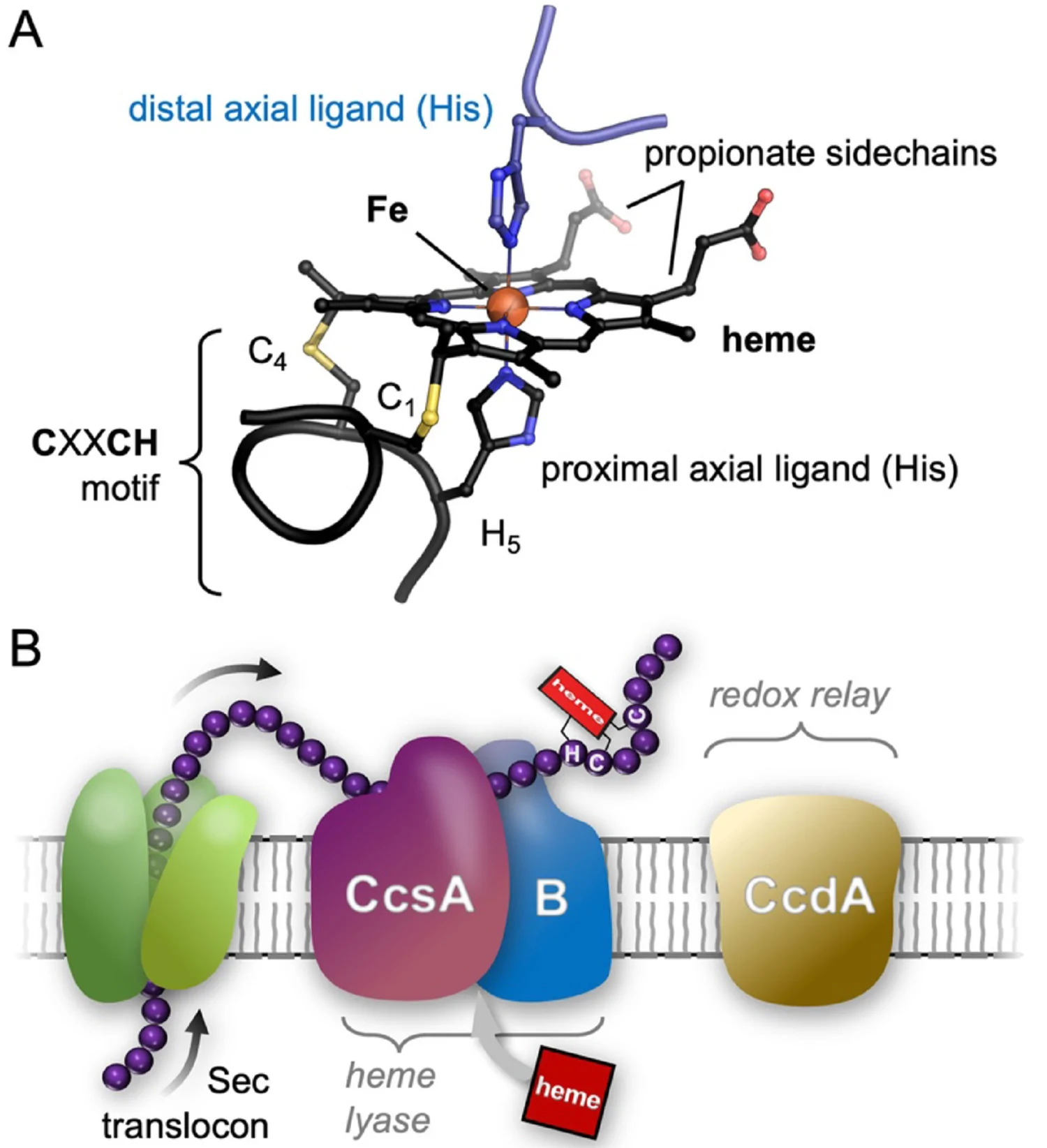
Maturation of cytochromes *c* by CcsBA. **A**) Structural features of heme *c*. The protoporphyrin IX moiety is covalently linked through its vinyl side chains to two cysteine residues (C_1_, C_4_) that form part of a Cx_*n*_CH binding motif. The terminal histidine of this motif forms the proximal axial ligand to the iron ion, while the distal axial ligand originates from another part of the protein and thus is variable. **B**) Cytochromes *c* are exported via the Sec system as apoproteins and subsequently scanned by a heme lyase complex that covalently attached heme groups to all binding motifs prior to protein folding. In system II, this heme lyase is the highly integrated CcsBA complex that also mediates heme translocation from the cytoplasm. It acts in conjunction with the CcdA protein that is required to reduce the internal disulfide bridge in the heme-binding motif as a prerequisite to heme attachment

Metalloproteins that utilize the cofactor heme (Fe-protoporphyrin IX) are summarily classified as cytochromes [1, 2]. Among different subfamilies, the cytochromes of type *c* are distinguished by the covalent attachment of one or multiple heme groups to conserved binding motifs within the amino acid sequence of the apocytochrome [3]. This covalent linkage results from an addition reaction of two cysteine residues within a conserved heme-binding motif of sequence –Cys–x_*n*_–Cys–His– to the two vinyl sidechains of the protoporphyrin IX ring (Fig. 1A). In most cases, the spacing of the cysteine residues is *n* = 2, but various exceptions are found, from *n* = 4 up to *n* = 15 [4]. The histidine residue within the binding motif typically serves as the proximal axial ligand to the heme iron. Here, too, exceptions exist, including binding motifs that replace His with a Lys in cytochrome *c* nitrite reductases [5, 6], or in cases where His forms part of the binding motif but does not ligate the metal in the mature protein [7]. Cytochromes *c* play essential roles in electron transfer reactions during respiratory and photosynthetic pathways, or act as biocatalysts for challenging multi-electron redox reactions [8]. As such, they are among the most widespread and diverse families of metalloproteins known to date [9]. Their outstanding hallmark is that they are utilized in evolutionarily ‘extracytoplasmatic’ compartments such as the bacterial periplasm, the mitochondrial intermembrane space or the thylakoid lumen of chloroplasts, or even as extracellular proteins [10]. Examples for the latter are secreted rubber oxygenases of the RoxA or LcpA type [11, 12], multiheme metal reductases [13, 14], or the remarkable, conductive pili of Geobacteraceae and *Shewanella* species that are prototypical biological ‘cables’ for long-distance electron transfer [15–20]. Here, the covalent linkage of heme ensures cofactor stability and facilitates the assembly of large protein complexes in environments where redox conditions and pH values can vary substantially.

The maturation of *c*-type cytochromes is a post-translational modification that requires highly specific maturases that attach the cofactors processively to every CxxCH motif in the apocytochrome chain [21]. This is the task of heme lyases, integral membrane protein complexes that receive the nascent protein chain directly after passing the Sec translocon [4, 22]. Indeed, in prokaryotes the maturation of cytochromes *c* is strictly linked to the Sec pathway, i.e. the export of unfolded peptides from the cytoplasmic space, rather than to the Tat pathway that translocates folded proteins through transient pore formation. To date, five different cytochrome *c* maturation machineries have been described, of which system I-III are the best characterized. System I, the Ccm system, prevalent in α- and β-proteobacteria, plant mitochondria, deinococci, archaea, and some γ- and δ-proteobacteria, uses a tripartite maturase assembly consisting of up to nine gene products (CcmA-I) with a distinct heme translocase (CcmABCD), the heme chaperone CcmE, and the lyase complex CcmFGH(I) that finally ligates the cofactor to the peptide chain. In system II, the Ccs system, found in β-, δ- and ε-proteobacteria, Gram-positives, aquificales and cyanobacteria, as well as in algal and plant chloroplasts, a simpler architecture is centered on the CcsAB complex that unifies the functions of heme translocation, apoprotein scanning and heme attachment into a single functional unit (Fig. 1B). System III is the mitochondrial system, where the required apocytochromes (mitochondrial cytochrome *c* and the *c*_1_ subunit of complex III) are encoded in the nucleus and imported into the intermembrane space via the TOM complex. System III heme lyase thus is a single peripheral membrane protein located on the intermembrane side of the mitochondrial inner membrane [23, 24]. Systems I and II differ markedly in complexity, but show high structural similarity in their lyase components, strongly suggesting substantial analogies in function.

A recent cryo-EM study has provided structural insights into CcsBA, a frequent fusion of the system II subunits, from *Helicobacter hepaticus* [25]. Here, two entirely unexpected conformational states became apparent in a single cryo-EM sample. In a ‘closed’ state, a heme group was buried in the transmembrane region of the lyase complex, in a position that was also observed in the system I CcmF subunit [26], while in the ‘open’ state a second heme was bound at a periplasmic site, accompanied by a large rearrangement that created a chamber for apocytochrome binding. These structures supported a possible pathway for heme movement within the complex and gave rise to a hypothesis how the lyase might coordinate the transport and ligation of the cofactor. The core lyase subunit CcsA, as well as CcmF and the heme translocase subunit CcmC, contain a conserved, tryptophan-rich WxWD or ‘heme-handling’ motif (HHM) and two histidine residues near the periplasmic side that in all cases are thought to mediate heme positioning and covalent linkage to the CxxCH motif [27]. However, several mechanistic questions remain unresolved, including how heme is maintained in the reduced state prior to ligation, how apocytochrome docks to the enzyme, what triggers the transition between closed and open states, and how electron transfer is managed during the process.

*C*-type cytochromes are integral to cellular energy metabolism and electron transfer, and their maturation requires an intricate multiprotein machinery linked to transmembrane protein export. System II, exemplified by CcsBA, integrates heme transport and ligation into a single complex, but despite recent structural breakthroughs key aspects of its mechanism remain unknown. In the present work we have studied the Ccs system from *Bacteroides thetaiotaomicron* that uses a fused *ccsBA* gene to mature its six *c*-type cytochromes. We report on the high-resolution cryo-EM structure of a W703C variant that was reported to trap heme by covalent attachment [25]. Form this analysis, we initially hoped to learn how a covalently attached heme group is situated in the two structural states observed in *Hh*CcsBA. However, while no such covalent linkage was observed, the structure contained non-covalently bound heme and provides critical mechanistic insight into heme handling, conformational regulation, and species-specific divergence of the System II machinery.

## Experimental procedures

### Cloning, protein production and isolation

The gene encoding the natural fusion protein *Bt*CcsBA was amplified via PCR from genomic DNA of *B. thetaiotaomicron* and subsequently cloned into a pETTSC vector, adding a C-terminal StrepTag(II). Recombinant *Bt*CcsBA^W703C^ was expressed in *Escherichia coli* C43 (DE3) cells [28] transformed with the pETTSC::Bth_CcsBA^W703C^ plasmid. No external hemin was added, and neither did we further enhance heme biosynthesis by addition of δ-aminolaevulinic acid. Cultures were grown in 3 L of Terrific Broth medium (TB; 12 g⋅L^–1^ of tryptone, 24 g⋅L^–1^ of yeast extract, 0.4%. (*v/v*) of glycerol, 0.017 M of KH_2_PO_4_, and 0.072 M of K_2_HPO_4_) at 37 °C until an optical density at 600 nm (OD_600_) of 1.8 was reached. Protein expression was induced by the addition of IPTG (final concentration 1 mM), and cells were incubated overnight under agitation at 30 °C. Harvested cells (55 g wet weight) were resuspended and subjected to standard membrane preparation protocols, yielding approximately 9 g of total membrane material. Membrane proteins were solubilized in buffer containing 50 mM Tris/HCl at pH 7.5, 300 mM NaCl, 10% (*v/v*) glycerol, and 0.03% (*w/v*) *n*-dodecyl-β-*D*-maltoside (DDM), and loaded onto a Strep-Tactin II affinity column (IBA) equilibrated in the same buffer.

To replace DDM with glycol-diosgenin (GDN) for downstream structural studies, detergent exchange was carried out on a column (Strep Tactin II), by applying a ten column volume linear gradient from buffer 1 (50 mM Tris/HCl pH 7.5, 300 mM NaCl, 10% glycerol, 0.03% DDM) to buffer 2 (50 mM Tris/HCl pH 7.5, 300 mM NaCl, 10% glycerol, 0.02% GDN) at a flow rate of 0.1 ml⋅min⁻¹. After reaching 100% of buffer 2, the protein was eluted by supplementation of 0.45 mM of desthiobiotin. The eluate was concentrated and further purified by size-exclusion chromatography (SEC) using a Superdex 200 increase 10/300 column (Cytiva) equilibrated in SEC buffer (20 mM Tris/HCl at pH 7.5, 150 mM NaCl, 0.02% GDN). Peak fractions containing CcsBA^W703C^ were pooled and used for subsequent cryo-EM analysis.

### Heme staining

In-gel heme staining was carried out with unstained SDS-PAGE gels according to published protocols [29]. In brief, the gel was incubated in a buffer containing 1.25 mM 3,3’,5,5’-tetramethylbenzidine, 30% (*v/v*) of methanol and 70% (*v/v*) of sodium acetate buffer at pH 5 for 30 min at 293 K. Subsequently, 30% H_2_O_2_ solution was added to a final concentration of 26 mM and incubated for approximately 10 additional minutes. The color reaction was stopped with a fixing solution consisting of 30% (*v/v*) of isopropanol in sodium acetate buffer at pH 5.0.

### Cryo-EM sample preparation and data collection

Purified CcsBA^W703C^ was concentrated to 5 mg⋅ml⁻¹ for cryo-EM grid preparation. Holey carbon grids (Quantifoil or UltrAUfoil R1.2/1.3, 300 mesh) were glow-discharged for 45 s at 15 mA using a GloQube Plus (Quorum Technologies) to increase surface hydrophilicity. A 3 µL aliquot of protein sample was applied to the grids under controlled environmental conditions (4 °C and 100% relative humidity) in a Vitrobot Mark IV (Thermo Fisher Scientific). Grids were blotted for 6 s using blotting force 5 (Whatman filter paper, GE Healthcare) and then plunge-frozen in liquid ethane cooled by liquid nitrogen. Frozen grids were clipped and transferred to a Titan Krios G4 transmission electron microscope (Thermo Fisher Scientific) operated at 300 kV and equipped with a Falcon 4i direct electron detector. Micrographs were recorded at a nominal magnification of 215,000×, corresponding to a calibrated pixel size of 0.572 Å. Data were collected using a defocus range of − 0.5 μm to − 2.4 μm.

### Data processing

A total of 23,299 movie frames were collected and processed using CryoSPARC v4.7. An initial set of ~ 10 million particle coordinates was extracted from ~ 14,000 motion-corrected micrographs and subjected to multiple rounds of 2D classification to discard false positives and improve particle quality. High-quality 2D class averages were subsequently used for template-based particle picking across the entire dataset, yielding 2.3 million particles. These particles were further refined through 2D classification, resulting in a subset of 534,335 particles used for ab initio reconstruction. From this subset, 192,319 particles contributed to an initial 3D reconstruction. Several iterative rounds of heterogeneous refinement and non-uniform refinement were performed, producing a structure at 4.08 Å resolution. This map was used as a reference for further heterogeneous refinement with particles picked from the complete dataset. Continued rounds of hetero and non-uniform refinement followed by local refinement led to a reconstruction at 3.47 Å resolution. Final clustering of exposures and additional non-uniform and local refinement steps improved the map to a final resolution of 2.91 Å.

### Structural modeling and refinement

The structural model for CcsBA was built in COOT [30], using an in-silico model generated with AlphaFold 3 as a starting point [31], iterating with real-space refinement in PHENIX to a final resolution of 3.19 Å (Supplementary Table 1) [32]. The model quality was assessed with MolProbity as part of the PHENIX suite of programs [33]. Figures were generated with UCSF ChimeraX [34] and PyMol (Schrödinger LLC.).

## Results and discussion

### Cryo-EM structure of CcsBA^W703C^ from *B. thetaiotaomicron*

**Fig. 2:**
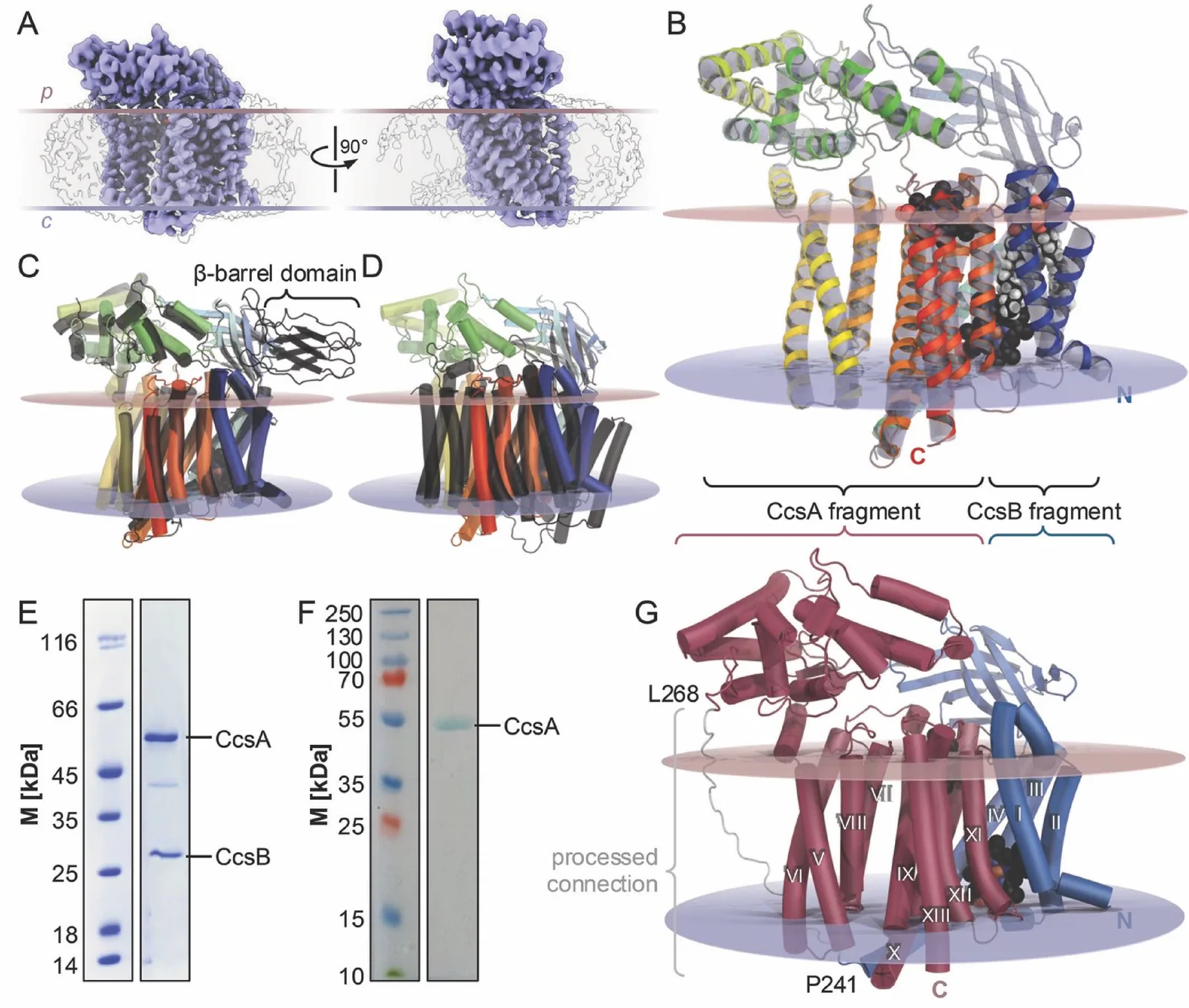
Architecture of *B. thetatiotaomicron* CcsBA. **A**) Coulomb maps from single-particle cryo-EM showing a front and side view of the CcsBA complex, as well as the detergent micelle. The position of the membrane is indicated, with the cytoplasmic boundary in blue and the periplasmic boundary in red. **B**) Cartoon representation of *Bt*CcsBA colored from blue at the N-terminus to red at the C-terminus. **C**) Overlay of *Bt*CcsBA with the structure of *H. hepaticus* CcsBA in the closed conformation (PDB 7S9Z). **D**) Overlay of *Bt*CcsBA with CcmF from *T. thermophilus* (PDB 6ZMQ), the core heme lyase CcmF of the cytochrome maturation system I. **E**) CcsBA was expressed from a single, fused gene, but SDS-PAGE shows that the protein is processed into two separate fragments. **F**) Heme staining of the SDS-PAGE shown in (E). A heme cofactor migrates with the larger CcsA fragment. **G**) The structure of *Bt*CcsBA shows two distinct chains corresponding to the fragments in (E). A connecting sequence between P241 and L268 is absent

Cryo-EM data were acquired on a Titan Krios G4 microscope operating at 300 kV. A total of 23,299 movies were collected and processed in CryoSPARC, resulting in a final reconstruction at 2.91 Å resolution using 143,981 particles (Supplementary Fig. 1). The local resolution ranged from below 2.6 Å in the transmembrane core to ~ 4.0 Å in the peripheral domains and the final model was refined to a resolution of 3.19 Å (Supplementary Table 1). The protein was embedded in GDN micelles and appeared as a monomer in a well-defined, closed conformation (Fig. 2A). The overall architecture of CcsBA^W703C^ from *B. thetaiotaomicron* is similar to that of the wild-type structure from *H. hepaticus* in the closed conformation (PDB 7S9Z) [35], comprising 13 transmembrane helices and large periplasmic domains in both the CcsA and the CcsB part (Fig. 2B). The core membrane-embedded regions align well with *Hh*CcsBA, with a root-mean-squared deviation of 1.8 Å for all atoms. However, the structures differ in the CcsB periplasmic domain, where *Bt*CcsBA^W703C^ lacked an additional β-barrel domain that forms an insertion into the shared periplasmic domain in the *H. hepaticus* structure (Fig. 2C). In-silico modeling and sequence alignments show that this domain is only present in a subset of CcsB proteins, but its exact role remains to be determined.

The bipartite heme lyase CcsBA is an integrated machinery for the posttranslational modification of apocytochrome peptides and is structurally similar to the core heme lyase CcmF of the cytochrome *c* maturation system I, for which we recently reported a crystal structure (Fig. 2D, PDB 6MZQ) [26]. However, it exhibits several relevant differences in topology and architecture that were not previously discussed. In organisms that carry a fused Ccs heme lyase, the *ccsB* portion consistently forms the N-terminal part of the *ccsBA* gene, preceding *ccsA*. Nevertheless, isolated CcsBA from *H. hepaticus* was reported early on to present as two distinct bands in SDS-PAGE, indicating proteolytic cleavage of the protein (Fig. 2E). We observed the same for *Bt*CcsBA^W703C^, with a 27 kDa CcsB fragment and a 50 kDa CcsA fragment. Only the latter was positive for heme-staining in SDS-PAGE (Fig. 2F), which was consistent with the previously suggested covalent attachment of a heme group in the analogous W828C variant of *Hh*CcsBA [25]. The available structures underline a further, unusual detail: Not only are fused CcsBA proteins posttranslationally cleaved, but the C-terminus of the CcsB fragment and the N-terminus of the CcsA fragment are situated on different sides of the membrane (Fig. 2G), and in the EM map a 24-residue segment from position 242 to 267 was undefined. It is not currently known whether this cleavage occurs on the cytoplasmic or periplasmic side of the membrane and which protease mediates it.

**Fig. 3:**
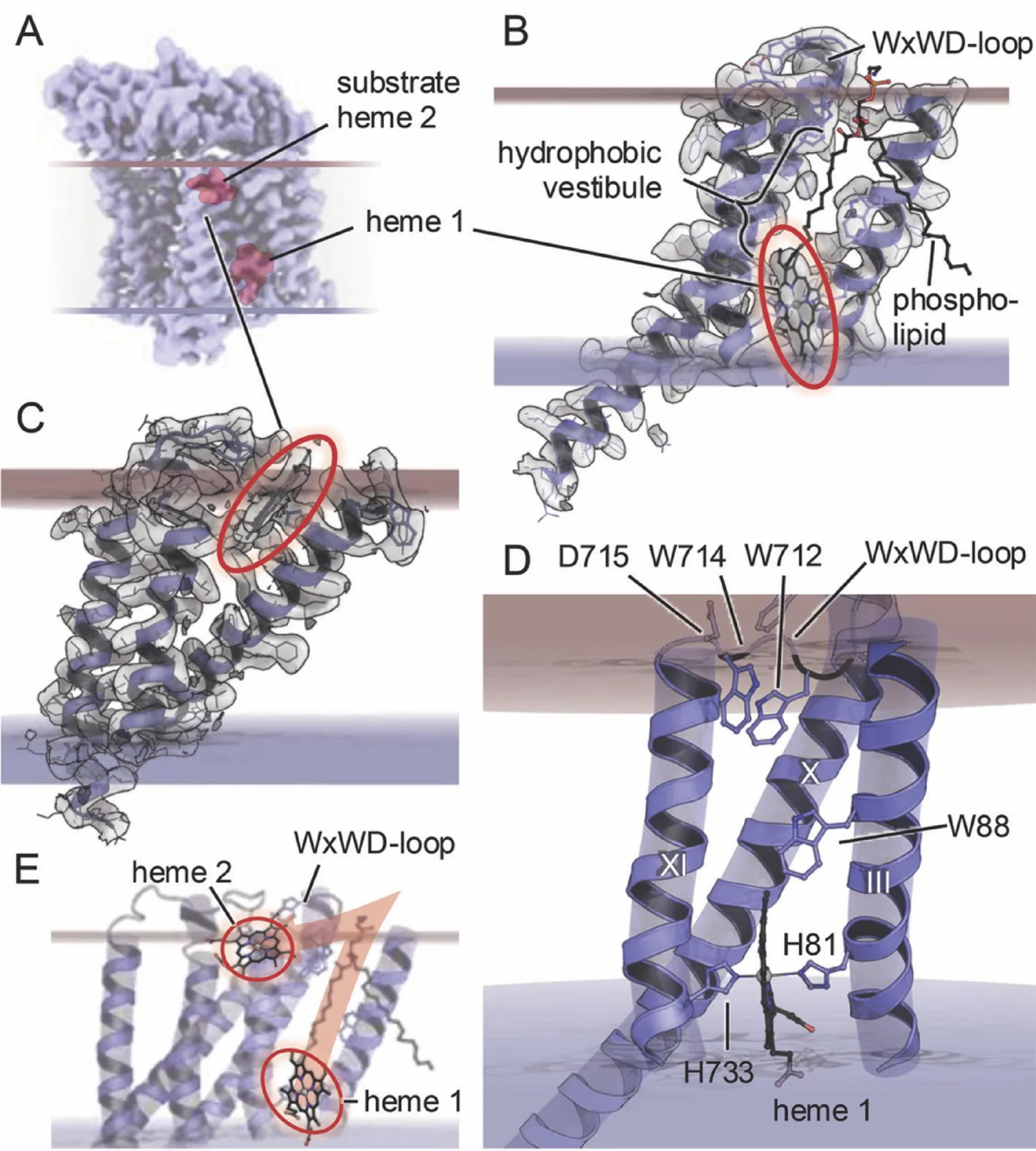
Heme binding and the heme translocation pathway in *Bt*CcsBA. **A**) Side view of the experimental map of *Bt*CcsBA W703C. Hemes 1 and 2 are bound at opposing sides of the lipid bilayer. **B**) Surroundings of the heme 1 binding site at the base of the heme translocation tunnel. The experimental map is shown for protein and heme but omitted for clarity for the bound phospholipid. **C**) The surroundings of heme 2 with the experimental map. Heme 2 is bound at the periplasmic face of the membrane. **D**) Detail of heme 1 with the axial ligands H81 and H733 and the WxWD loop at the periplasmic side, situated above the heme translocation channel. **E**) Contrary to the structurally related system I heme lyase CcmF (Fig. 1D), heme 1 in CcsBA is translocated across the membrane through the unobstructed transport channel to interact with the WxWD loop that mediates a 90° rotation of the cofactor, placing it into the heme 2 site for attachment to the apocytochrome chain

The more complex Ccm system distributes various subtasks of heme maturation to additional modules [22, 36, 37]. The CcmABCD complex acts as a heme translocase, flipping heme to the outer leaflet of the cytoplasmic membrane and handing it off to the heme chaperone CcmE [38]. CcmE then transports heme to the heme lyase complex CcmFGH(I), where CcmI scans and directs the apocytochrome chain, while CcmH and CcmG contain redox-active disulfides to retain heme-binding motifs in the reduced state [39]. In the Ccs system, heme transport, apoprotein scanning and heme attachment are integrated into a compact, multifunctional and dynamic membrane protein complex, while the redox functionality may not be required for the anaerobic or microaerophilic organisms that employ system II. The structural analyses of CcmF from *Thermus thermophilus* [26] and of *Hh*CcsBA have confirmed the similarity of both enzymes that was suggested by their amino acid sequences. Both heme lyases share a heme group bound at the cytoplasmic side of the membrane and designated heme 1 in the following (Fig. 3A). It is situated below a hydrophobic vestibule that can be occupied by a phospholipid (Fig. 3B, Supplementary Fig. 2A). Both also feature the WxWD heme-handling motif, and an additional, conserved set of histidine residues (H635 and H774 in *Bt*CcsBA) on the periplasmic side of the complex. In BtCcsBA, these histidine residues serve as axial ligands to coordinate a second heme, the substrate heme 2 (Fig. 3C). However, the role of the membrane-integral heme group 1 seems to differ substantially. In CcmF, the substrate heme is delivered by CcmE and inserted into the hydrophobic vestibule above heme 1 via a lateral opening to the outer leaflet of the membrane [26]. Heme 1 was suggested to then reduce the substrate heme, facilitating its release from CcmE and handover to the adjacent WxWD motif (Fig. 3D) [22]. No such chaperone is required in CcsBA, where heme 1 is likely directly translocated across the membrane to reach the WxWD motif between TM helices X and XI through an internal connection to the adjacent hydrophobic vestibule and from there on to the substrate heme position (Fig. 3E). From the structures of *Hh*CcsBA it has become obvious that this internal channel that in CcmF is obstructed by two Trp residues [26] is sufficiently large to allow the passage of heme 1 toward the extracytoplasmatic side of the membrane. Reporting on a cryo-EM structure of the heme translocase CcmABCD, we have previously suggested that the role of the WxWD motif in the CcmC subunit of this complex is in receiving the heme group and mediating a rotation by almost 90° [40]. In CcmC, this serves to position a vinyl group of heme for attachment to the cofactor chaperone CcmE, and in CcsBA the very same kind of rotation would serve to transfer the cofactor from the end of the transmembrane channel to the binding site of heme 2. This supports the hypothesis that the specific function of the WxWD heme-handling motif indeed consists of rotating the cofactor while passing it through an opening between two helices that form part of a conserved four-helix bundle, encompassing the C-terminal helices 9–12 in CcsBA (Fig. 3D) [40].

### Coordination of heme 2 and configuration of the WxWD motif

**Fig. 4:**
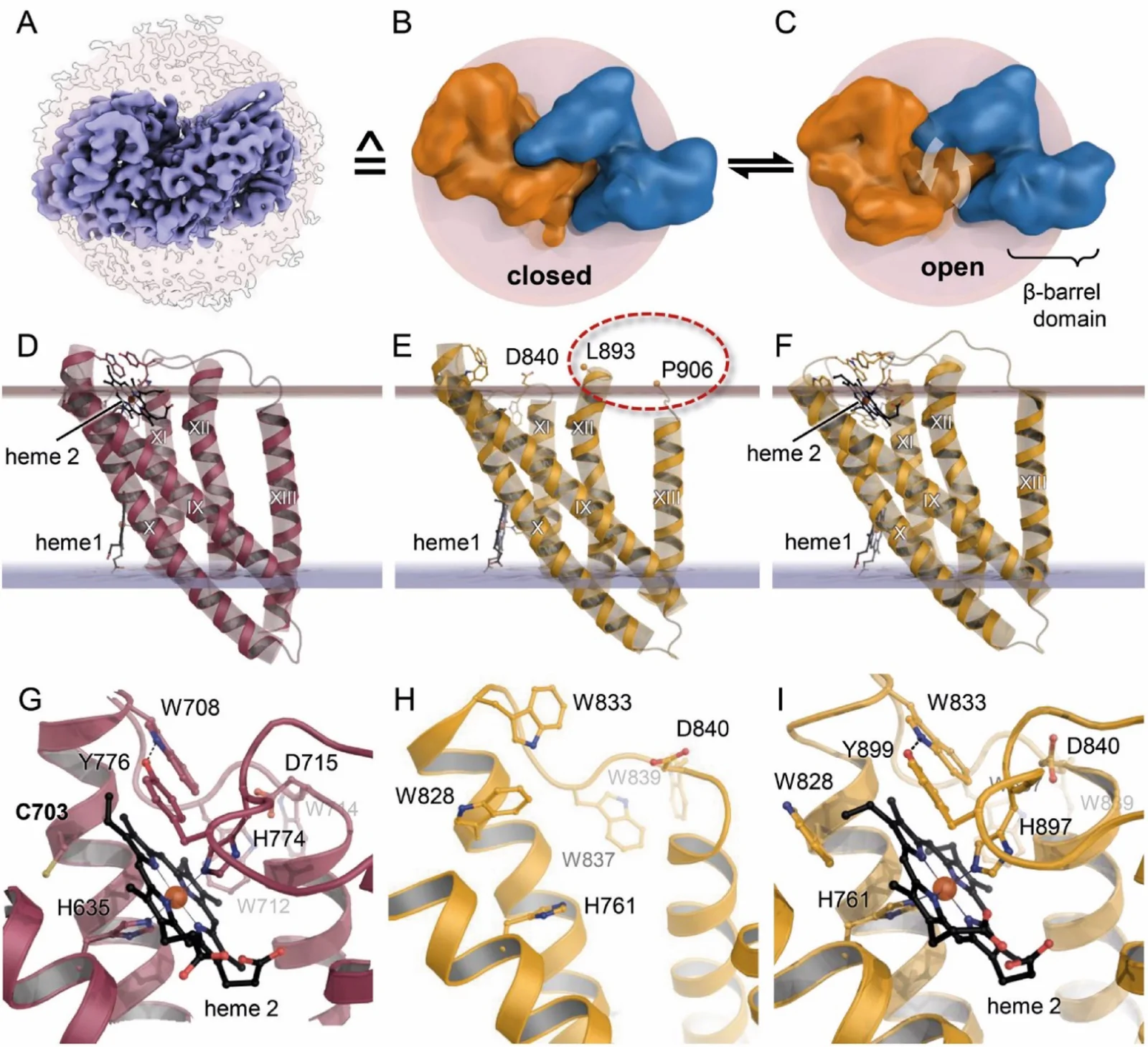
The W703C variant of *B. thetaiotaomicron* CcsBA. **A**) Top view of the experimental map of *Bt*CcsBA and the surrounding GDN micelle. **B**) Structurally, the *Bt*CcsBA structure corresponds most closely to this closed state of *Hh*CcsBA (PDB 79SZ) but contains heme 2 in its periplasmic binding pocket. **C**) In the open state, the two periplasmic domains of *Hh*CcsBA move further apart (PDB 7S9Y). **D**) Detail of helices IX-XIII of *Bt*CcsBA with bound hemes 1 and 2. The distal histidine ligand to heme 2 forms part of the extended loop connecting helices XII and XIII. **E**) The same region as in panel (D) shown for the closed state of *Hh*CcsBA. **F**) The same region as in (D) for the open state of *Hh*CcsBA, with bound heme 2. **G**) Detail of heme 2 coordination in *Bt*CcsBA^W703C^. **H**) The empty heme 2 pocket of open-state *Hh*CcsBA. Note that W828 blocks the binding pocket. **I**) The heme 2 pocket in closed-state HhCcsBA. W828 and W833 sandwich the cofactor

The cryo-EM structure of *Bt*CcsBA^W703C^ (Fig. 4A) corresponds well to the closed-state structure of *Hh*CcsBA (Figs. 2C and 4B), which in turn was found to alternate with an open state (Fig. 4C), through a transition that largely consists of a rocker-switch like, relative movement of the two four-helix bundles of the CcsA fragment, helices V-VII and IX-X plus XII-XIII (Fig. 2G) [25]. Based on work by Kranz and co-workers, the *Bt*CcsBA^W703C^ variant was designed to covalently trap heme at the presumed active site of the complex [25]. This was indeed the case, but despite the presence of heme 2 (Fig. 4D), *Bt*CcsBA remained in the closed conformation, almost perfectly retaining the structure of *Hh*CcsBA in the closed state, where this heme was absent (Fig. 4E). Heme 2 binding in *Hh*CcsBA was observed exclusively in the open-state structure (Fig. 4F), in a mode and position that very closely resembled the one observed in *Bt*CcsBA^W703C^. On closer inspection, the heme 2 moiety in the mutant structure was well resolved and coordinated axially by residues H635 and H774, the predicted covalent bond to residue C703 was absent (Fig. 4G). The introduced cysteine residue adopted a side-chain orientation consistent with that of the native tryptophan in the closed conformation, suggesting that the smaller size of the side chain alone was sufficient to permit heme access without a conformational rearrangement that may be required in the wild-type protein. In the closed-state structure of *Hh*CcsBA, the corresponding W828 is oriented towards the heme-binding pocket, likely preventing ligand binding (Fig. 4H). This supports the hypothesis that W703 acts as a steric gate that needs to rotate to accommodate the heme group passed along through the WxWD motif. In contrast, in the open-state structure of *Hh*CcsBA (PDB 7S9Y, Fig. 4C), heme 2 is sandwiched by the side chains of two tryptophan residues (W828 and W833) that correspond to W703 and W708 in *Bt*CcsbA^W703C^. In the structure of *Bt*CcsBA^W703C^, W708 is present but W703 has been replaced by cysteine, which is sufficient to allow for the binding of heme 2 in the open conformation of the complex. Other residues in the surrounding of the ligand are also more consistent with the open state seen in *H. hepaticus*, despite the overall closed conformation of the complex. Notably, W708 forms a hydrogen bond to tyrosine Y776 of *Bt*CcsBA^W703C^ (Fig. 4G), which is mirrored by W833:Y899 in the open state of *Hh*CcsBA (Fig. 4I). The lack of a covalent bond to the CcsA fragment seemingly contradicts the positive heme stain we and others have observed (Fig. 2F). However, it may be assumed that this integral membrane protein, like many others, does not fully unfold in SDS-PAGE, as is frequently seen by a smaller apparent molecular weight than what its amino acid sequence would suggest. In the structure, the observed hybrid state suggests that, in the absence of steric hindrance from W703, a partial activation of the active site is possible without full domain movement. Our data imply that W703 not only occludes the active site in the closed conformation (Fig. 4H) but may also function as the conformational switch that triggers the observed, major structural rearrangement between states in *Hh*CcsBA when heme is provided via the WxWD motif.

### Functional consequences of the W703 substitution

The observation that only the closed conformation was captured, despite the presence of P-heme, reinforces the notion that the open state is not strictly required for heme translocation but is critical for ligation. In the wild-type protein, W703 likely plays a dual role: first, it physically blocks the active site in the closed state, and second, it triggers the opening of the periplasmic domain upon rotation. In the *Bt*CcsBA^W703C^ variant, the smaller cysteine permits passive heme binding but fails to elicit domain opening, thus effectively decoupling translocation from structural activation. This insight refines the working model for CcsBA activity. Heme delivery via the WxWD motif can occur in the closed state via the internal channel, but the successful covalent attachment to apocytochrome *c* may require full domain opening—likely triggered by heme-induced WxWD loop rearrangement or by the interaction with the apocytochrome substrate, for which access likely is far easier in the open state. The absence of a covalent heme–C703 bond further suggests that precise spatial coordination is required for vinyl–thiol linkage, and that such positioning is not achieved in this variant.

## Conclusion

The cryo-EM structure of CcsBA^W703C^ from *Bacteroides thetaiotaomicron* reveals that heme can be accommodated in the active site without triggering global conformational change, challenging (or refining) assumptions about the coupling between heme binding and structural activation in System II cytochrome *c* maturation. These findings underscore the role of W703 as a steric and regulatory element and point to organism-specific structural adaptations in the periplasmic domain. Together, these data support a model in which heme translocation can proceed in a structurally closed state, while ligation requires an open conformation that is coordinated by a tryptophan-triggered conformational switch.

## Supplementary Information

Below is the link to the electronic supplementary material.

Supplementary Material 1

Supplementary Material 2

## Data Availability

The structural model and map for BtCcsBAW703C have been deposited with the Protein Data Bank and the Electron Microscopy Data Base with accession codes PDB 25VK and EMD-80405.

## Abbreviations

Ccm: Cytochrome c maturation
Ccs: Cytochrome c synthesis
HHM: Heme-handling motif
