# Supplementary material for: Heme handling in the system II heme lyase CcsBA from Bacteroides thetaiotaomicron
Source: J Biol Inorg Chem. 2026 Aug 25;31(6):567–76. doi: 10.1007/s00775-026-02171-y (PMC13645880; doi:10.1007/s00775-026-02171-y)

Seifermann *et al.*

SDS-PAGES; Figure 2E,F

Figure 2E

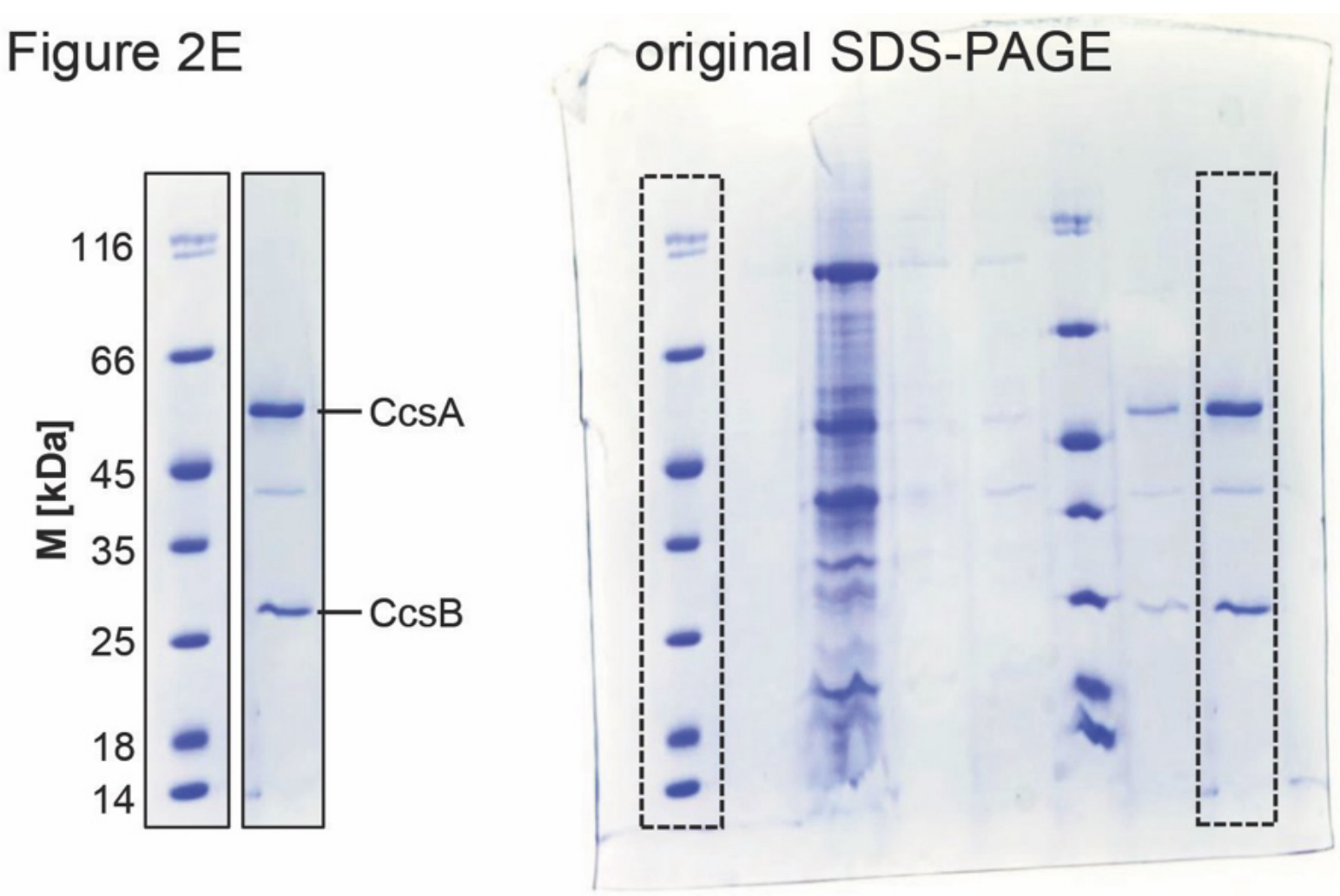


Figure 2F

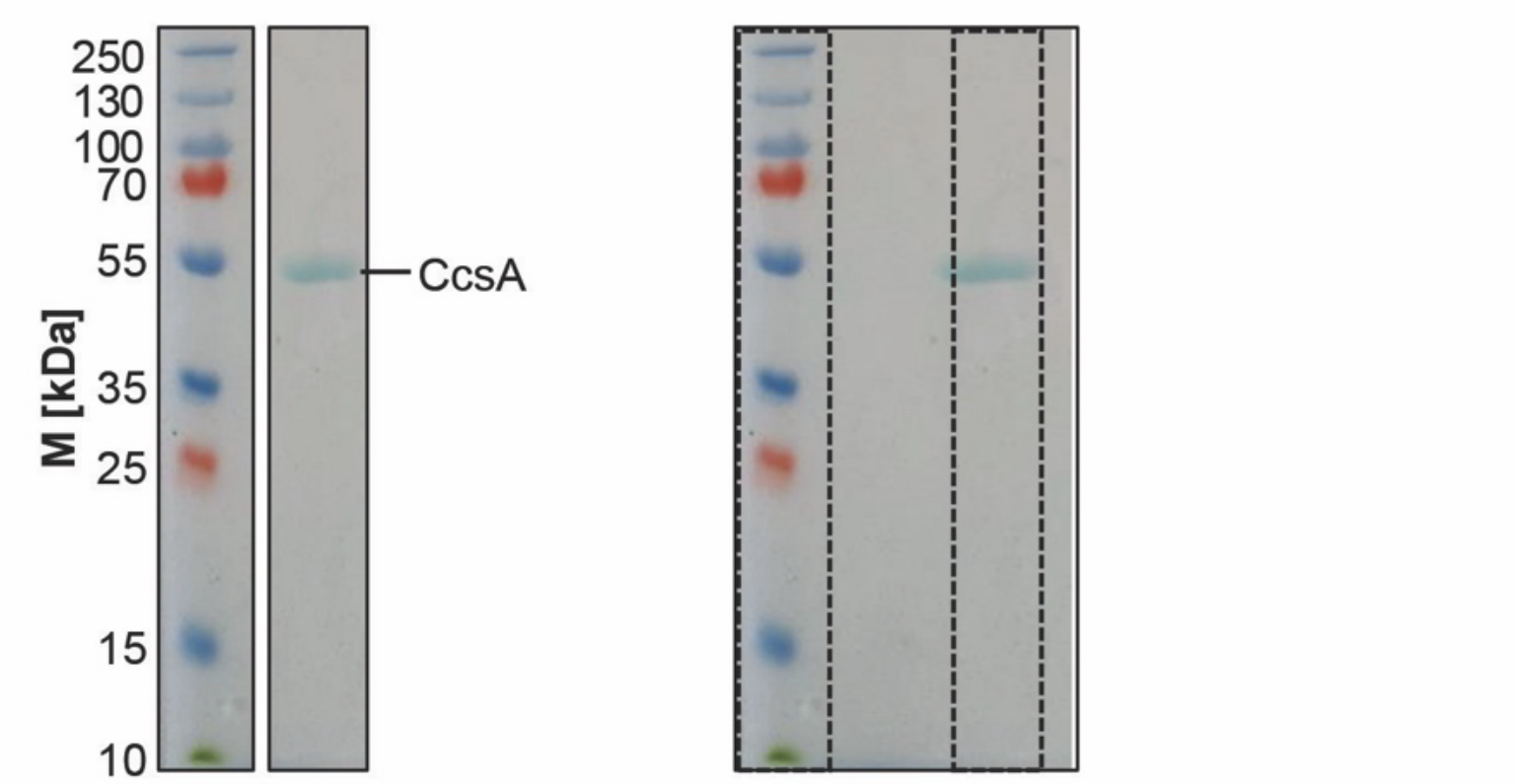

Supplement: Supplementary file 1 [file 775_2026_2171_MOESM1_ESM.pdf]
