# Supplementary material for: Heme handling in the system II heme lyase CcsBA from Bacteroides thetaiotaomicron
Source: J Biol Inorg Chem. 2026 Aug 25;31(6):567–76. doi: 10.1007/s00775-026-02171-y (PMC13645880; doi:10.1007/s00775-026-02171-y)
Supplement: Supplementary file 2 [file 775_2026_2171_MOESM2_ESM.docx]

**SUPPLEMENTARY INFORMATION**

**Supplementary Figure 1:** Cryo-EM workflow. **A**) Representative micrograph. **B**) Selected 2D classes from 23,299 recorded movies. **C**) Refinement workflow and particle numbers for 2D and 3D classification. **D**) Angular distribution of particles for the final refinement round of the map. **E**) Fourier shell correlation (FSC) curves for *Bt*CcsBA using the gold standard threshold of 0.143 to determine the overall resolution of 2.91 Å.

**Supplementary Figure 2:** Stereo images for representative Coulomb density maps. **A**) The region surrounding heme 1 and the bound phospholipid, as well as the WxWD motif. The density for the bound phospholipid has been omitted for clarity. Stereo rendering of main text Fig. 3B. **B**) Binding of the substrate heme 2. Stereo rendering of main text Fig. 3C.

**Supplementary Table 1.** Cryo-EM data collection, refinement, and validation statistics.

| **Data Collection** |  |
| --- | --- |
| PDB ID | 25VK |
| EMBD ID | EMD-80405 |
| microscope | Titan Krios G4 |
| detector | Falcon 4i |
| magnification | 215,000 |
| voltage (kV) | 300 |
| exposure (e^–^/Å^2^) | 40 |
| defocus range (µm) | –1.0 to –2.0 |
| raw pixel size (Å) | 0.572 |
| number of movies | 23,299 |
| initial particles | 3,764,142 |
| imposed symmetry | *C*_1_ |
| final particle number | 143,981 |
| resolution (Å) | 2.91 |
| FSC threshold | 0.143 |
| **Refinement** |  |
| Map pixel size (Å) | 0.7627 |
| Model resolution (Å) | 3.19 |
| FSC threshold | 0.5 |
| Model composition |  |
| Non-hydrogen atoms | 6,418 |
| Protein residues | 786 |
| Ligands | 3 |
| *B* factors (Å^2^) |  |
| Protein | 84.23 |
| Ligand | 64.72 |
| R.m.s. deviations |  |
| bond lengths (Å) | 0.003 |
| bond angles (°) | 0.645 |
| Validation |  |
| MolProbity score | 1.48 |
| clash score | 6.65 |
| poor rotamers (%) | 0.00 |
| Ramachandran plot |  |
| favored (%) | 97.44 |
| allowed (%) | 2.56 |
| disallowed (%) | 0.00 |
